# HIV control: Is getting there the same as staying there?

**DOI:** 10.1371/journal.ppat.1007222

**Published:** 2018-11-01

**Authors:** Philip Goulder, Steven G. Deeks

**Affiliations:** 1 Department of Paediatrics, University of Oxford, Oxford, United Kingdom; 2 HIV Pathogenesis Programme, Doris Duke Medical Research Institute, University of KwaZulu-Natal, Durban, South Africa; 3 Department of Medicine, University of California, San Francisco, California, United States of America; University of Pittsburgh, UNITED STATES

## Abstract

In this brief review and perspective, we address the question of whether the immune responses that bring about immune control of acute HIV infection are the same as, or distinct from, those that maintain long-term viral suppression once control of viremia has been achieved. To this end, we describe the natural history of elite and post-treatment control, noting the lack of data regarding what happens acutely. We review the evidence suggesting that the two clinical phenotypes may differ in terms of the mechanisms required to achieve and maintain control, as well as the level of inflammation that persists once a steady state is achieved. We then describe the evidence from longitudinal studies of controllers who fail and studies of biologic sex (male versus female), age (children versus adults), and simian immunodeficiency virus (SIV) (pathogenic/experimental versus nonpathogenic/natural infection). Collectively, these studies demonstrate that the battle between the inflammatory and anti-inflammatory pathways during acute infection has long-term consequences, both for the degree to which control is maintained and the health of the individual. Potent and stringent control of HIV may be required acutely, but once control is established, the chronic inflammatory response can be detrimental. Interventional approaches designed to bring about HIV cure and/or remission should be nuanced accordingly.

## Introduction

Identifying the mechanisms by which the host can naturally control HIV or simian immunodeficiency virus (SIV) has long been a priority for immunologists. These mechanisms might be leveraged to develop novel interventions to prevent HIV transmission, control HIV in the absence of therapy (a “remission”), or even fully eradicate the reservoir (a “cure”) [1]. Towards this end, groups around the world have recruited and characterized those rare individuals who maintain near-complete control of the virus in the absence of antiretroviral therapy (ART). Two distinct clinical phenotypes exist: those who naturally control the virus without any treatment (“elite” controllers) and those who do so but only after receiving prolonged ART (“post-treatment controllers”).

Most studies of elite and post-treatment controllers focused on those individuals who are recruited during a period of long-term host-mediated control. Although often unstated, these studies assume that those mechanisms that maintain control are the same as those that initially brought the virus under control. This assumption is convenient but has limitations. In this brief review and perspective, we challenge this assumption and argue that the optimal immune response needed to achieve control differs from that needed to maintain control. Untangling these mechanisms might be needed before we can develop effective prevention and curative interventions.

## Natural history of elite and post-treatment control

The natural history of individuals who are destined to fully control their virus in the absence of therapy (elite control) or after interrupting therapy (post-treatment control) remains poorly defined. This is particularly true during the immediate post-infection or post-interruption period in which the virus likely replicates in the absence of a fully formed host response. Because most controllers are identified long after the acute viremic phase has resolved, the kinetics of HIV replication and the immediate host response are poorly understood.

### Elite control

Depending on the definition, approximately 0.5% to 1% of untreated individuals eventually achieve elite control [2]. Although the chronic steady-state biology of HIV control has been well studied [3–6], little is known about the acute phase. Many, if not most, of these individuals express the HLA-B*57:01 allele [7, 8]. HLA-B*57:01 has been reported to be under represented in people presenting with acute infection, suggesting low levels of acute viremia and at least partial control of the virus during this time [9]. In the prospective United States Department of Defense HIV Natural History Study and the European-based Choices, Attitudes, and Strategies for Care of Advand Dementia at the End-of-Life (CASCADE) cohorts, the level of viremia in early infection was lower in controllers than noncontrollers, but data during the acute phase were lacking [10, 11]. In the prospective Prediction of Muscular Risk in Observational conditions (PRIMO) cohort, eight controllers were identified during early infection (median 2.2 months after infection) and were found on average to have low levels of viremia [12], but there was substantial variability and no one was diagnosed in acute phase when peak viremia would have occurred. Low levels of viremia during the acute and/or early phase have also been reported in several case reports and small cohorts [13–19]. Although data from the acute phase of peak viremia are scarce, the collective data suggest that peak viremia during the acute phase is likely lower than that in more typical infection. Elite control is likely driven in part by a favorable host response that is active during the earliest stages of the infection.

Given the challenges of studying acute infection in elite controllers, animal models may be needed to advance this story. Elite control has been observed in SIV-infected macaques, particularly those carrying the MHC class I allele Mamu-B*08 or Mamu-B*17. Approximately 50% and 25% of Mamu-B*08-positive and Mamu-B*17-positive macaques, respectively, achieve control following infection with the highly pathogenic SIVmac239 [20, 21]. In humans infected with HIV, the presence of protective HLA class I molecules such as HLA-B*57 and HLA-B*27 are less predictive of control. This differential effect of protective alleles may be due to the nature of the infection; macaques are experimentally infected with a single clone, whereas humans are naturally infected by a huge diversity of viruses.

In the macaque model, viremia during the acute phase did not differ between those animals with or without a protective class I allele type; monkeys with Mamu-B*08 had high-level viremia before achieving control [20]. Transient but high-level viremia was also observed in other models of SIV control [22]. Therefore, the SIV model suggests that the host immune response emerges quickly during acute infection but perhaps not until after a transient period of high viremia. Whether this is true in humans remains unknown. The monkey model will likely be needed to untangle the complex virus–host interactions that occur prior to the establishment of elite control.

### Post-treatment control

Since the first identification of the post-treatment control phenotype [23], the field has struggled with how to define and study this relatively rare clinical phenotype [24]. There is even an ongoing debate as to whether post-treatment controllers are simply elite controllers destined to control their virus even without a period of ART [25, 26]; indeed, in the Short Pulse Anti-Retroviral Therapy at Seroconversion (SPARTAC) cohort, the frequency of at least transient control in the absence of therapy was not too different from the degree of transient control among those who were treated early and then stopped [25].

Arguing for their legitimacy are the observations that the two clinical phenotypes of post-treatment control and elite control are distinct in a number of important characteristics. The frequency of post-treatment control among those starting and stopping ART in early infection is higher than one might expect for natural control (approximately 5% to 20% versus 0.5% to 1%, depending on definitions) [23, 27–32]. The reservoir declines over time in some post-treatment controllers but is remarkably stable in elite controllers [27, 33]. HIV-specific cluster of differentiation (CD)8+ T-cell responses are generally low in post-treatment controllers but high in elite controllers [27, 34, 35]. CD8+ T-cell activation (defined by co-expression of CD38 and HLA-DR) is lower in post-treatment controllers than elite controllers [27]. The distribution of class I HLA alleles may also be different, as described below.

As with elite controllers, there is a dearth of information regarding what happens during the immediate post-treatment period when responsibility for virus control shifts from ART to the host immune response. Perhaps the best data come from a recent study of a therapeutic vaccine in which post-treatment control was relatively common in the control group [28]. In this study of people who had started ART early, 4 of 15 (26%) subjects in the placebo arm and 2 of 14 (14%) in the vaccine arm had at least partial control for at least 16 weeks off therapy, with 2 in the placebo arm exhibiting sustained control. Acute post-ART spikes in viremia were initially observed in most of these controllers, with levels that were often but not universally low (100 to 10,000 copies RNA/mL). Similar findings have been observed in a recent multi-cohort study (the Control of HIV after Antiretroviral Medication Pause [CHAMP] cohort) [36].

A critical question regarding post-treatment control is the degree to which ART fundamentally switches the balance in favor of the host immune response. This question can be addressed in part by comparing the pre-ART viral load with the level of post-ART control. The pre-ART levels of viremia in post-treatment controllers have also been variable, with evidence from two cohorts suggesting that a low viral load in acute infection predicts a low viral load post-ART, although the effect was modest [12, 30]. In contrast, in the Viro-Immunologic Sustained Control After Treatment Interruption (VISCONTI) cohort pre-ART viremia was reported to be much higher in people destined to become post-treatment controllers than those destined to become elite controllers [27]. Similarly, in the largest study of post-treatment controllers identified to date (the CHAMP cohort), the level of viremia prior to ART was similar in individuals who controlled post-ART compared to those who failed to control post-ART [32]. In contrast, the reservoir size (often estimated by frequency of HIV DNA levels in circulating CD4+ T cells) has been reported to be low during ART in those who eventually achieve post-treatment control [27, 34, 37, 38]. These observations, which are admittedly based on small numbers and incomplete data, suggest that ART can shift the host–virus balance towards one that favors the host.

## Mechanisms of elite control likely differ from those of post-treatment control

A clue to the central mechanisms underlying elite control of HIV infection is the high frequency of “protective” HLA molecules such as HLA-B*57 and HLA-B*27 and low frequency of “disease-susceptible” HLA molecules such as HLA-B*35 [8, 39]. In contrast, in the VISCONTI post-treatment controller cohort, HLA-B*35 was highly prevalent, and HLA-B*27 and B*57 were notably infrequent [27]. In the SPARTAC study, the study cohort was a mixture of subtype-B–infected male Caucasians and subtype-C–infected female Africans, and in this heterogeneous group, one or more disease-susceptible HLA alleles (defined as HLA-B*35:01 or HLA-B*07:02 for subtype-B and HLA-B*18:01 or HLA-B*58:02 for subtype-C) were also found in the post-treatment controllers, whereas there was no clear enrichment for the classic protective alleles [25]. Classically defined protective alleles were rare in another recent cohort of post-treament controllers [38]. Among post-treatment controllers, the ability of HIV-specific CD8+ T cells to inhibit viral replication is modest—and similar to that of typical non-controllers—but significantly lower than that of elite controllers. This prompts the question of whether those controlling HIV after treatment are doing so because of an immune response that does not rely on generation and persistence of the immunodominant HLA-B-restricted CD8+ T cell specificities observed in many elite controllers.

The differential distribution of HLA alleles in post-treatment control and elite control may provide hints as to why levels of T-cell activation or inflammation are lower in the former group, as has been described [27]. HLA molecules have unique binding affinity for the leukocyte immunoglobulin-like receptors (LILRs), which are believed to play a regulatory role in dendritic cell function. HLA alleles associated with elite control (HLA-B57 and HLA-B27) enable the generation of aggressive, highly activated and immunodominant CD8+ T-cell responses in acute infection [9, 40–42]. These HLA molecules have low binding affinity for the LILRB2 receptor and hence induce less regulation of dendritic cells and ultimately greater activation of T cells. In contrast, HLA-B*35 has high binding affinity for LILRB2 and hence likely induces a greater degree of dendritic cell tolerization and less robust induction of CD8+ T-cell responses [43].

Collectively, these HLA stories argue that elite control is maintained via sustained and highly potent HIV-specific CD8+ T-cell activity that is part of a highly activated and inflammatory immune response, whereas post-treatment control is maintained via a less inflammatory response that may be less dependent on these pathways. This is perhaps not surprising as the pathway needed to achieve initial control is fundamentally different in these two very distinct clinical presentations: the amount of “work” that the immune system needs to perform in getting the virus under control is likely much higher in elite control (in which a naïve system is generating a response during very high levels of HIV replication) versus post-treatment control (in which an exposed system might be primed to respond well before high viremia emerges).

Less is known about the mechanisms associated with post-treatment control. Putative immunologic correlates of control include T-cell function (particularly CD4+ T-cell responses), natural killer (NK) cell function (noncytolytic activities have been observed) and low T-cell activation (lower than that observed in natural control and during ART) [27].

**Fig 1:**
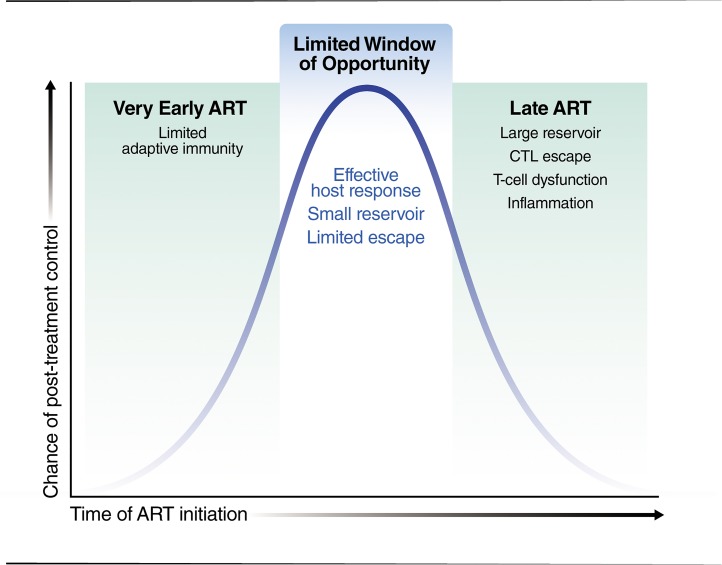
The time ART is initiated may prove to be critical determinant of post-treatment control. Treatment of “hyperacute” HIV infection (Fiebig 1) may prevent the development of an effective immune response. During a subsequent treatment interruption, the virus will rebound rapidly, and there will be limited chance for post-treatment control. In contrast, a delay in treatment for too long will result in a generation of escape mutants, a large and difficult-to-control reservoir, and a damaged immune system. ART, antiretroviral therapy; CTL, cytotoxic T lymphocyte.

Perhaps the most consistent correlate of post-treatment control is a low reservoir [27, 38]. Based on mathematical modeling, it has also been argued that the early initiation of ART prevents the establishment of a large reservoir and that, in this setting, a less effective host response may be sufficient to maintain durable control after therapy is interrupted [44]. That the early initiation of ART blunts the establishment of a large reservoir is now well accepted [45–48]. Early therapy also protects immune function [49, 50], but if started too early, it might prevent the generation of effective HIV-specific T-cell memory [42]. If therapy is delayed too long, escape mutants are generated [51], the reservoir size becomes less manageable, and the immune system is irreversibly damaged. Consistent with these results, treatment of “hyperacute” HIV infection (Fiebig 1) fails to cause post-treatment control [52, 53] whereas treatment of long-term established infection is also rarely protective. Early ART (but not too early) may be needed (Fig 1). The timing of ART in VISCONTI and other post-treatment controllers largely supports this concept.

## Limitations inherent in cross sectional studies of elite and post-treatment controllers

The vast majority of mechanistic studies regarding HIV control in people are cross sectional in nature. These studies have well-known limitations. Specifically, it is difficult, if not impossible, to determine cause and effect. The study of host genetics and outcome partially addresses this concern, as the genes were present before the infection and distinguishing cause and effect is straightforward.

During low-reservoir states, either in elite control or post-treatment control, the activity of the host response is expected to be low even if it is actively controlling the virus, complicating the interpretation of any observations made during steady state. This dilemma is well illustrated by studies of extraordinary control of the virus in which the host responses were barely detectable even though they were assumed to be active and effective [54]. The CD8+ T-cell response during elite control is characterized as one with greater proliferative potential [55]; this suggests that the immune system is largely at rest and waiting to expand as needed when infrequent bursts of replication occur. Nonactivated central memory CD8+ T cells that target diverse epitopes and have the capacity to expand and differentiate are also enriched in elite controllers [56, 57]. These cells are not abundant or particularly active, suggesting that they are present in vivo but not being stimulated systemically. The nature of these responses is distinct from the massive, high-magnitude, and apparently highly activated response typical of “hyperacute” HIV infection [41, 42].

There is no reason to assume that the pathways that gain control of HIV are not the same as those that maintain control, but it is almost certainly true that once the control is gained, the activity of these responses will decline, making it challenging to infer how the immune system gained control in the first place. Still, longitudinal studies of the chronic phase will likely prove informative in defining how best to maintain control (and perhaps avoid any collateral damage).

## Can we learn anything from controllers who eventually progress?

Chronic inflammation during elite control has been well-established [58–61]. These inflammatory pathways likely contribute to immune dysfunction [62] and perhaps the development of cardiovascular disease [63, 64].

Virologic rebound or progression among elite controllers is uncommon [11, 65–68] and poorly understood. In one multinational observational study of individuals followed from seroconversion (CASCADE), virus control (<500 copies RNA/mL) occurred in a small minority (1.4%) but once achieved was maintained in most (the probability of maintaining control over 20 years was 0.74) [11]. In a multicenter study of controllers (Collaboration of Observational HIV Epidemiological Research Europe [COHERE]), progression was rare and was predicted by a low CD4/CD8 ratio and intermittent viremia [67]; similar findings were noted in a French study [68]. In smaller pathogenesis-oriented studies, heightened inflammation and T-cell activation predated and often predicted virologic progression [17, 69, 70].

The rates of virologic progression may be higher in post-treatment controllers than elite controllers. In the CHAMP cohort, post-treatment controllers were identified from a collection of studies in which individuals were followed from the time they interrupted through at least 24 weeks. The majority of those who controlled for at least 24 weeks (and hence met the definition of a post-treatment controller) exhibited virologic rebound within three to four years [32]; this rate is much higher than that reported in most elite controller cohorts. In the SPARTAC cohort, there was a trend suggesting that those achieving control in the absence of ART were more likely to maintain control than those who achieved it after an ART interruption [25].

The collective natural history studies suggest that post-treatment control is more common than elite control but perhaps less durable. Two scenarios may explain these trends. Both clinical phenotypes might involve the same mechanism, but a weaker one is sufficient to achieve post-treatment but not elite control. Alternatively, distinct mechanisms underlie these clinical phenotypes, and those involved in post-treatment control work well in an acute setting (when ART is interrupted) but not in a chronic setting (when control needs to be maintained indefinitely).

## Greater immune control of viremia but more rapid disease progression among females

The natural history of untreated HIV infection differs by sex. In untreated infection, the viral load set points in females are 0.33 to 0.78 log_10_ copies RNA/mL lower than in males [71]. Women are 5-fold more likely than men to be elite controllers [65]. Females are also more likely than males to clear hepatitis C virus (HCV) in the absence of treatment [72]. Mechanistically, it has been argued that plasmacytoid dendritic cells (pDCs) in females produce substantially more interferon-alpha in response to stimulation by toll-like receptor 7 (TLR7) ligands such as HIV-1 and other single-stranded RNA viruses [73] (perhaps, as recently argued, because TLR7 is on the X chromosome and is expressed at higher levels in females than males) [74]. Although type I interferon has potent antiviral activities in the acute setting [75], too much interferon-alpha, or too much of the wrong subtypes of interferon-alpha, can lead to immune hyperactivation and its well-described detrimental consequences [76–81].

Although women may control HIV more often than men and have lower viral load set points, women progress to AIDS at a more rapid rate for any given viral load than men [82]. High levels of immune activation for any given level of viremia may account for this apparent accelerated progression in women [73]. Also, women have a greater tendency for viral rebound after having achieved elite control [65]. Chronic inflammation has also been a consistent predictor of virus rebound among established controllers [17, 69, 70] and could contribute to virologic progression through multiple mechanisms [83], as described below.

Collectively, these observations argue that immune response during acute infection is more aggressive and effective in females compared with males. This does not come without a cost, however, because this heightened inflammation during the chronic phase in females is associated with higher risk of losing virus control and progressing to AIDS. The consistent observation that women have much higher rates of a number of autoimmune diseases (e.g., systemic lupus erythematosus [SLE], autoimmune thyroid disease, myasthenia gravis, rheumatoid arthritis, multiple sclerosis, Sjogren syndrome, scleroderma) [84] is broadly consistent with these observations. Theoretically, the potent anti-HIV response in females is more effective in achieving initial control than that observed in males, but the possibly less inflammatory (or more tolerant) response in males is more effective in maintaining control.

## Pediatric versus adult HIV infection: Immunotolerance versus immune aggression

In pediatric HIV infection, the ability of the immune system to control HIV via an effective CD8+ T-cell response is largely neutered by the immunotolerant environment of early life [85, 86]. Consequently, viral loads are higher in children than adults; indeed, the median viral loads are approximately 1.5 log_10_ higher in young children than in adults at the equivalent time after infection [87, 88]. Elite control, as defined by having an undetectable viral load by standard assays, is rare in perinatally infected children but has been reported [89, 90]. Post-treatment control is also rare but has been reported [35, 91, 92].

In contrast to elite control, long-term non-progression is relatively common. At least 5% of untreated HIV-infected children maintain normal-for-age CD4 counts through 5 to 10 years of life with high levels of viremia (“viremic nonprogressors”) [93, 94]. This poorly researched phenotype has been reported in adults but at frequencies much lower than those observed in children [95–97]. Among children, nonprogression during high viremia has been associated with low levels of T-cell activation in children. As children mature and become adolescents, the steady-state levels of viremia decline while the levels of immune activation increase [93].

The children versus adult comparison is hence similar to the male versus female comparison among adults. The apparently more immunotolerant environment of young life seems to prevent the immune system from achieving stringent control of HIV replication yet at the same time protects against chronic inflammation, CD4+ T-cell loss, and disease progression.

## Pathogenic versus nonpathogenic SIV infection

SIV infection of monkeys provides additional support for our model. The natural hosts for SIV have low levels of immune activation and disease progression despite persistently high viral loads [98, 99], a phenotype that is similar to human viremic nonprogressors [97]; indeed, these two share a number of similar biologic profiles [95, 96]. Elite control in the natural hosts for SIV is rare (most animals have viral loads well above 10,000 copies RNA/mL) although not uncommon in experimental models such as rhesus macaques [20], in which the virus stimulates a profound inflammatory response and causes rapid disease progression.

## The double-edged sword effect of HLA-B*57 and HLA-B*27

HLA-B*57:01 is the single human genetic polymorphism most strongly associated with elite control of HIV infection [7]. The mechanism for this protective effect has yet to be fully defined. Many have argued that HLA-B*57, HLA-B*27, and other protective alleles present multiple highly conserved epitopes for CD8+ T-cell recognition, escape from which results in a significant fitness cost to the virus [100–102]. Perhaps the clearest evidence that epitope specificity is important is the observation that distinct protective class I alleles in rhesus macaques (Mamu-B*17) and humans (HLA-B*27:02) target the identical immunodominant epitope (Nef IW9 IRYPKTFGW in SIV and IRYPLTFGW in HIV) [103, 104] despite independent evolution of human and rhesus macaque MHC class I. Similarly, the two protective alleles Mamu-B*08 and HLA-B*27:05 share a similar peptide binding groove and can present the identical epitopes [20]. It has also been reported that enhanced HIV-specific CD8+ T-cell function—as defined by proliferative capacity, cytotoxic activities, and the production of multiple cytokines [5, 6]—has been a consistent predictor of virus control, arguing that the inherent functional capacity of the immune response in addition to the epitopes targeted is a key factor. This raises the possibility that HLA-B*57:01 and other protective alleles somehow stimulate the generation of highly functional CD8+ T cells directly and independent of the epitopes targeted [105].

Along these lines, HLA-B*57:01–restricted CD8+ T-cell response during acute infection is notably more potent and sustained than other HLA-restricted responses [8, 106, 107] Also, HLA-B*57:01 positivity is associated with the clearance of HCV [108], particularly in North America [109].

The protective effects associated with HLA-B*57:01 positivity may come at a cost, however, because this allele is associated with hypersensitivity reactions, most notably to abacavir [110]. Similarly, HLA-B*27:02 and HLA-B*27:05 are protective against HIV but also associated with a long list of autoimmune disorders [111]. Many of the genes associated with protection against HIV (including but not limited to HLA-B*57:01 and HLA-B*27) are associated with higher risk of developing psoriasis among those with HIV and in the general popuation [112].

There is no unifying mechanism to explain the apparent connection between HLA, immune control of HIV, and autoimmunity. A number of possibilities have been proposed, including shared epitopes [112], unique thymic maturation pathways that result in a T cell receptor (TCR) repertoire that is broader and often more autoreactive [107] (and hence more able to respond to escape mutants, another factor apparently associated with control [113–115]), an ability to evade regulatory T-cell suppression [116], and a lack of dendritic cell regulation due to poor binding avidity of HLA-B*57 and HLA-B*27 with LILRB2 [43], as described above.

A recent set of observations regarding the complex interaction between HLA molecules and NK cell receptors provides another potential mechanism by which particular HLA molecules might substantially modulate the impact of the innate immune response. KIR3DL1 is expressed on NK cells and, when engaged, inhibits the function of these cells. HLA molecules bind killer cell immunoglobulin-like receptors (KIRs) and influence NK cell function in complex ways. HLA-B*57:01 is the strongest binder to KIR3DL1 of all HLA allotypes tested [117]. People expressing both HLA-B*57:01 and specific allotypes of KIR3DL1 are more likely to control HIV than those expressing just HLA-B*57:01 [118]. Co-expression of the KIR3DL1 variant, I47V, further enhances the protective effect of HLA-B*57:01 [119]. The mechanism for this interaction between HLA and KIR molecules is unknown. One possibility is that NK cells expressing high levels of KIR3DL1 are licenced to kill HIV-1-infected target cells directly [120] and are unleashed by altered HLA-B*57:01 and/or peptide expression arising in the HIV-infected targets [121]. Alternatively, NK cells regulate antiviral CD8 T-cell responses, the presence of NK cells expressing inhibitory KIRs improving CD8+ T-cell function [122]. A further possibility is that KIRs expressed on CD8+ T-cell surface may compete with TCRs that recognize the same peptide–HLA-B*57:01 complex. Alterations in any of these pathways could conceivably result in collateral effects and higher or lower risk of autoimmune and hypersensitivity response.

Regardless of the mechanism, the HLA-B*57 and HLA-B*27 riddles once again illustrate that, although the capacity to generate a potent inflammatory response might be helpful in clearing or controlling a pathogen, this intrinsic capacity could also lead inflammation-associated harm.

## Concluding remarks

**Fig 2:**
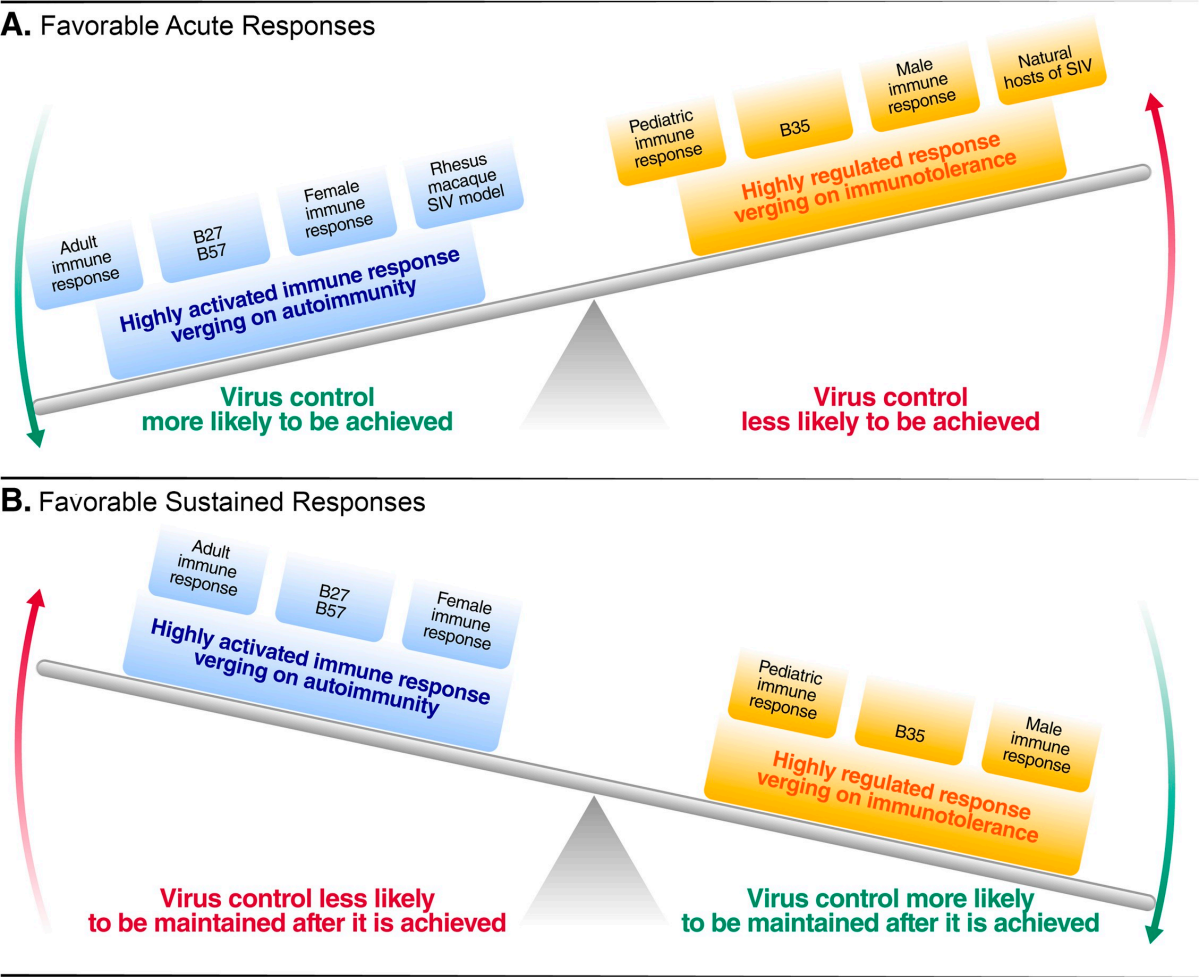
Favorable immune responses in order to achieve control of viremia in acute infection and in order to maintain control once achieved as in post-treatment control. (A) Favorable immune responses to achieve control in acute infection. (B) Favorable immune responses to maintain virus control once achieved. SIV, simian immunodeficiency virus.

As has been consistently demonstrated in studies of SIV and HIV, potent and sustained immune responses to chronic pathogens cause collateral damage. Those who are more likely to achieve elite control are in general more likely to experience disease progression, as shown by our comparisons of women versus men, adults versus children, and natural versus experimental models of SIV (Fig 2). The most robust predictors of virus control in untreated disease are also consistently associated with autoimmunity and other inflammatory disorders. Longitudinally, once elite control is achieved, those individuals who exhibit higher levels of inflammation are more likely to lose control over time.

Why might chronic inflammation during periods of virus control eventually cause virologic rebound? Theoretically, inflammation may enhance virus production and/or result in greater numbers of susceptible targets for the virus (activated CD4+ T cells), leading to more virus spread [83]. Alternatively, the persistent inflammation can result in a sustained counter-regulatory responses and inhibition of T-cell function, a phenomenon that has been observed in cancer tissues and is now being targeted by a variety of immune therapies [123]. Up-regulation of programmed cell death protein 1 (PD-1) and other checkpoint receptors, induction and expansion of immunosuppressive cell populations (T regulatory cells, myeloid-derived suppressor cells), and the expression of potent immunosuppressive pathways (indoleamine 2,3-dioxygenase, interleukin 10 [IL-10], and transforming growth factor beta [TGFß]) are all associated with HIV-mediated inflammation. All are now being modified in experimental oncology, and many are being targeted in HIV cure strategies [124]. Similarly, activation of TLR-mediated type I interferon signalling is currently being pursued as a means to reverse latency and stimulate sustained immune responses [125, 126].

The recent success of canakinumab, an inhibitor of the inflammatory IL-1ß pathway, in preventing cardiovascular disease progression and cancer provides proof of concept that chronic inflammation is harmful and can be targeted therapeutically [127, 128]. The fact that blocking this pathway also increased the risk for infectious disease complications highlights the “double-edged sword” problem that we argue here will always need to be addressed in the context of an HIV cure or remission strategy [129].

The HIV cure agenda is expanding. Many, if not most, of the approaches now in the clinic seek to achieve long-term control of a replication-competent reservoir (a “remission”). Many of these approaches have relied on studies of elite and post-treatment controllers as the inspiration and even as a road map, but perhaps a more nuanced approach may be needed. It seems that a more holistic approach might be better achieved by examining those states of control that are more durable and less inflammatory in nature.
